# The Recommendations of the Royal Commission on Lunacy and Mental Disorder
*Read at a Meeting of the Bristol Medico-Chirurgical Society, December 8th, 1926.


**Published:** 1927

**Authors:** J. V. Blachford

**Affiliations:** Late Medical Superintendent, Bristol Mental Hospital


### THE RECOMMENDATIONS OF THE ROYAL COMMISSION ON LUNACY AND MENTAL DISORDER.*

BY

J. V. Blachford, C.B.E., M.D.,

*Late Medical Superintendent, Bristol Mental Hospital.*

---

The Report of the Royal Commission on Lunacy and Mental Disorder has been issued, and it is the purpose of this paper to discuss the conclusions and recommendations as to certification, and treatment without certification, of patients suffering from mental disorder, and the difference in the methods of procedure necessitated by the alterations in the present Act. Treatment without certification, as a voluntary boarder, is already possible for a patient who places himself in an institution for treatment provided he knows what he is doing, where he is, and the nature of the contract, and he may leave the said institution at any time by giving twenty-four hours' notice in writing; but this only applies to those who can afford to pay the fees of a private mental hospital or licensed house, and so advantage cannot be taken of this method by one who would be a State-aided patient.

Under the Act of 1890 patients requiring certification are divided into two classes, private and pauper.

* Read at a Meeting of the Bristol Medico-Chirurgical Society, December 8th, 1926.

For the former, the law requires that there shall be a petition, accompanied by a statement of particulars, giving name, age, occupation, etc., two medical certificates (by men in no way connected with the patient, or each other, who must examine the patient separately), and a magistrate's order for detention, though there is no necessity for the magistrate to see the patient. In the case of the pauper the patient is taken before the magistrate by a relieving officer, and only one medical certificate is required, but the magistrate must see the patient. In addition, there is the "urgency order," by which a private patient may be admitted to a licensed house on one medical certificate and the order of a relative, and which only holds good for seven days.

The recommendations of the Commission are as follows :—

(1) That all patients be divided into two classes, voluntary and involuntary.

The voluntary system will apply to all cases, State-aided as well as private.

In the case of State-aided cases the visiting committee will be responsible for admitting or refusing, but in cases of urgency the Medical Superintendent may admit on his own discretion and report the case to them as soon as possible.

The notice to take their discharge is to be seventy-two hours instead of twenty-four as at present.

(2) The involuntary cases will be divided into two classes, those in whom recovery is probable in or under six months, and cases which in the opinion of the medical man will require longer; there will be no distinction between pauper and private patients.

Those likely to recover in a reasonable time will be placed under restraint by a "provisional treatment order," to obtain which a relative, friend or public official will sign a petition for an order, and will take it with a statement of particulars of the patient's name, age, etc., and a disclosure of his property if any, and one medical certificate (in which it must appear that there is a fair chance of the patient's recovery within a reasonable period) to a Justice of the Peace, specially appointed, who must see the patient either at the patient's house or in an institution; and the magistrate, if he thinks fit, may make an order for provisional treatment which will authorise his detention in an institution for one month. If at the expiration of the month the medical superintendent of the institution is of opinion that the patient has not recovered he will give a certificate to that effect, and call in a Justice of the Peace, who may extend the order for another five months. If at the end of that time, six months in all, he is still of opinion that the patient is not well enough to be discharged, the patient must be certified in the ordinary way.

To certify a patient—in other words, to obtain a reception order—the petitioner must take his signed petition with statement of particulars, disclosing among other facts the patient's property if any, and two medical certificates by independent men (who may consult together), stating in the case of a person who has not been detained under a provisional treatment order that in their opinion the patient is not likely to recover within a reasonable time, to a Justice of the Peace specially appointed, who must see the patient, and, if he thinks fit, may issue an order for his detention. As regards the "urgency order," no change has been suggested.

The Commission also suggests that means should be taken for the more effective protection of medical men and others in the *bona-fide* discharge of their duties, and suggests amendment of Section 330 of the Lunacy Act, 1890, so as to provide that no person shall be liable to civil or criminal proceedings unless he has acted in bad faith or without reasonable care, and that any proceedings commenced against such person *shall* be stayed upon a summary application to the High Court or Judge in Chambers, unless the Court or Judge is satisfied that there is substantial ground for believing that such act was done in bad faith or without reasonable care.

Section 330 Sub-sec. 2 of the 1890 Act says: "If any proceedings are taken against any person for signing, etc., such proceedings *may*, upon summary application to the High Court or a Judge thereof, be stayed." The substitution of the word "shall" for "may" will be welcomed by all having anything to do with the certification or detention of the insane, and had it been inserted in Section 330 of the 1890 Act would almost certainly have prevented the unfortunate litigation which occurred so recently.

These, then, are the suggested alterations in the Lunacy Act regarding the early treatment and certification of patients.

(1) As regards the voluntary boarders, we must all agree that provided efficient means are employed to prevent the misuse of public funds, the suggestion is excellent; we have all seen many borderland cases that could hardly be classed as certifiable, and who, had they had the chance to put themselves under treatment voluntarily, would have done so. In some of these future certification would have been avoided, and in others the tragedy of a suicide escaped. But the difficulty I foresee in dealing with these is that a large number of the applicants must be drawn from among the constituents, or their friends, of the members of the Committee considering their case, thus placing these gentlemen in a very invidious and unfair position; and as there is a general tendency to increase the comforts and decrease the discipline in our mental institutions, besides increasing the number on parole, a time may arrive at no distant date when these may become more or less hotels for the unemployed or unemployable, especially in times of stress, the tariff being paid by the public. The abolition of the distinction between the pauper and the private cases, placing them all under the same procedure as regards certification, supplies a long-felt want. The present system is an anachronism, cumbersome, and at times very unfair.

When we come to deal with the Provisional Treatment Order it is difficult to discover anything in its favour. In the first place the Commissioners, Medical Superintendents, and medical men throughout the country for years have been insisting that there is no stigma in certification, that there is no more disgrace in a man becoming insane than in his having pneumonia or cardiac trouble; now, by introducing a method, the obvious purpose of which is to avoid the name of certification, they are admitting a fact which they have been denying, or at any rate are perpetuating a fallacy that for years they have been trying to dispel. In the second, can anyone tell me the difference between being detained under an Order for Provisional Treatment and under an Order for Detention? In both there must be a medical certificate, a statement of particulars divulging all the patient's private affairs. Both patients must be seen by a Justice of the Peace. In both the civil rights and liberty are lost until recovery. And in both the patient must be discharged if and as soon as he recovers.

Thirdly, there is the expense. This will be very materially increased both for those who enter private institutions and those who contribute to the maintenance of public asylums; the multiplicity of certificates, transport of judicial authorities, and court expenses mean something, and as copies of all orders and papers have to be sent to the Board of Control immediately, a considerable increase in the clerical staff of institutions will be necessary. With the income and other taxes at their present height, and no prospect of reduction, with local rates soaring and out of all proportion to the ability of many to pay them, it seems strange that a body of men should deliberately set out to increase public expenditure when so little benefit to the patients concerned is to be expected.

I have discussed only that part of the report which deals with the admission of patients, that being the only portion likely to be of interest to practitioners. I have, however, read most of the other suggestions, and can only say that, although it contains a number of alterations which will be very welcome, on the whole it is a disappointment. Having had conferences, correspondence, enquiries, and at last a Royal Commission, its authors must be congratulated on having upheld the best traditions of our Government Departments in having expended the greatest amount of energy with a minimum result. In the words of the old fable: "A mountain was in labour, sending forth dreadful groans, and in the neighbourhood there was the highest expectation. After all, it brought forth a mouse."

